# Student skills need to evolve to match our new AI society

**DOI:** 10.1016/j.patter.2024.101062

**Published:** 2024-10-11

**Authors:** Brent A. Anders

**Affiliations:** 1 American University of Armenia, Yerevan, Armenia

## Abstract

There are new realities in society and the workplace that necessitate the evolution of student skills. AI now creates highly usable text, requiring students to shift their focus to different skills such as editing, AI literacy, and critical thinking so that they may effectively work with AI and succeed in the modern world.

## Main text

Now that a year has passed since my article “Is using ChatGPT cheating, plagiarism, both, neither, or forward thinking?”,^1^ and in considering ongoing AI developments, ever increasing use-cases in business and everyday life, I see some improvements in educational institutions’ policies and some growing understanding of generative AI by academia. Yet, this is all much too slow and not enough. A much-needed shift must occur in how we view and use AI in academia. Popular but now outdated thoughts on generative AI, yet one’s I still hear many teachers and professors state, are AI can’t write very well, I can always tell when a student has written an essay with it, AI is just hype and isn’t really being used by business, students are already using AI so they don’t need to be taught AI literacy, and the only thing AI is good for is to cheat. The reality is that AI now creates highly usable written output that many businesses are starting to integrate into their work processes. This necessitates that academia realize that the skills needed by graduating students have evolved and that the use of AI, as eluded in my previous article, is forward thinking.

Students will always need to know how to write well, but more and more, most people will be using other skills as they work with generative AI to both create and modify writing as part of their jobs. Though using AI to create text might have been seen as “cheating” in the past, it is now quickly becoming a needed work skill. For the last 2 years, students have been sharing stories with me about how they are being asked in job interview and at places where they are already working at what type of AI skills they have. This correlates with information I have heard from colleagues internationally, and recommendations from Yahoo Finance and Upwork dealing with the importance of developing AI skills and placing it on your resume, regardless of the job field.^2^^,^^3^ The skills and subskills that are now being more heavily used include text editing, AI literacy, and critical thinking. For students to be able to effectively and efficiently succeed in this new reality and workplace, these new writing skills and capabilities must be emphasized within all of academia.

### Text editing skills

Generative AI is now capable of creating high-quality text with no discernable difference from what a human can write, but a human should always be in the loop to ensure quality assurance and create the best final product.^4^ Effective text editing skills are important and will become even more so as AI use in the writing process further increases. Word processors such as MS Word, Google Docs, and supportive software such as Grammarly all now have generative AI tools within their systems. Students should still be required to write essays and reports on their own, but now, given the improving AI technology, this ability should be used more as foundational knowledge in the development of students’ editing ability. Students need to develop a capability to know what “right” looks like when they review writing and use editing subskills such as flow, grammar, organization, comprehension level, validity, and consistency. Additionally, students need to be able to effectively modify text to improve tone and feel along with creativity and emotional connection-intelligence to best address the needs of the intended audience. Although these skills and subskills are needed in the writing process in general, their level of development and implementation are different when focused on editing AI output as opposed to initial creation without the use of AI.

Assignments and assessments need to incorporate specific and direct evaluation of students’ text editing skills in order to ensure that these skills can be fully transferred to the work environment. Using AI shouldn’t be seen as cheating, but instead its use should be integrated into effective means of creating written products. Process is important as well, so what I’ve done is use a mixture of in-class work and discussion, along with assignments such as having students use generative AI to create an entire business report and then go through and effectively edit it. This type of pedagogical evolution is needed to ensure students fully develop the necessary editing experience to succeed. A vital aspect of developing these editing skills will be properly understanding and developing AI literacy skills in order to best work with AI in general.

### AI literacy

In writing my 2023 book, *The AI Literacy Imperative*, I found that is vital for everyone to develop foundational knowledge of AI overall so as to then be able to develop greater abilities in using it effectively and ethically.^5^ I categorize AI literacy into four components: awareness (knowing that AI is all around and affecting society in different ways), capability (knowing AI limitations and how to use AI via prompt engineering), knowledge (knowing that all people now have free access to generative AI and are using it in different ways and knowing about data safety and security with information given to an AI), and critical thinking (bias, ethics, environmental considerations, overreliance).^5^ Although all aspects of AI literacy are important and have a synergistic effect on people’s abilities, the key concepts of capability and critical thinking need to be focused on in education to best develop students’ capacity to work with AI.

In working with students, I’ve found that although many of them now have some capability in using AI, they lack true prompt engineering skills, the ability to effectively write questions, tasks, queries to the AI in order to get the most useful and correct output-product. There is a major difference in using a simple prompt such as “write a report on cryptocurrency” versus using an advanced prompt:

Please assume the role of a professional cryptocurrency investor and researcher. Create a three-page report that explains what cryptocurrency is and why it might be a good investment. Provide specific examples within the last 5 years on how it has been used within different countries to help stabilize its economy. Be sure to also address two dangers of cryptocurrency and how they are being overcome. I need the report as part of an assignment for my university economics class. Use the provided rubric so that the report you create falls within the established guidelines. Do you understand?

In my experience, most students have no idea that an advance prompt formula consists of the following.

Task: what do you want the AI to do (“Create a … report”)
Instruction: specifics on how you want the AI to accomplish something (“three-page … explains what cryptocurrency is and why it might be a good investment … use the provided rubric”)
Context: the perspective or role you want the AI to take or personify (“assume the role of a professional cryptocurrency investor and researcher”)
Reasons: why or background to help the AI better create a response (“I need the report as part of an assignment for my university economics class”)
Clarification: giving the AI an opportunity to ask questions (“Do you understand”)
Refine: interacting with AI is a conversation, follow-on questions or modifications can continue as necessary until full information is obtained or the best product is created

Students need to be directly taught prompt engineering to include its subskills: vocabulary, logic, patience, persistence, analytical skills, problem-solving skills, attention to detail, creativity, and formulation. Students additionally need to know different prompting techniques such as one-shot prompting, providing an example response or file to help the AI understand, and zero-shot chain of thought, adding the statement “Let’s think step by step” to help see the AI’s process and help the AI reflect and stay on task.^5^^,^^6^^,^^7^

### Critical thinking

I see a lot of students that either try to memorize everything or think that AI will simply do everything for them; both of these students are wrong. Knowledge is now easily accessible through the Internet. What society and business need and want are people that are critical thinkers so that they can use AI to accomplish more. Critical thinking is now a must and a needed aspect of AI literacy. This deals with mindful awareness of what the AI is doing and how it is responding. It means that the user of AI is paying attention to the results, verifying information, asking follow up questions when needed, using multiple points of view, taking aspects such as bias and ethics into account, and realizing issues posed by AI to include environmental factors and dangers of overreliance.^4^ This importance was highlighted in a *Fortune* article from leadership consultant and Navalent CEO Ron Carucci, entitled “In The Age Of AI, Critical Thinking Is More Needed Than Ever”: “Regardless of how you ultimately end up using AI, sharpening your own critical thinking and problem solving skills will ensure that you can continue to provide value and grow in your chosen career path”^8^ (para. 24).

A friend of mine, who teaches at the University of Florida, shared how their educational institution is working to integrate AI within the entire curriculum; this is a prime example for all of academia.^9^ Academia must ensure that students develop critical thinking as part of their AI literacy development, especially with regards to writing. We can each do our part to ensure that students develop these skills. In teaching my professional communication course, I have found that students can readily develop these skills through discussion and realistic assignments. Problem-based learning along with roleplay and simulations, served as an excellent mechanism to help students gain knowledge, capability, and real experience with this needed skill.

A major facet of AI literacy critical thinking deals with different ethical considerations. What are the laws associated with AI’s use within a given work environment? What are the policies and guidelines? What information can and cannot be given to the AI? Is it always appropriate to use AI? Do customers need to be informed if they are talking to an AI or if the content created that they are reading was made with AI? Are all AI’s (ChatGPT, Gemini, Claude, and many others) the same when it comes to data safety and security? Does AI use excessive electricity, and if so, what can be done to address the issue? What are the dangers associated with AI and what actions can be taken? These are the type of important and relevant questions I give my students and that all modern students need to be able to address.

I recently heard of an A+ student who was denied an internship at a major company because when asked what type of AI literacy skills she had, she couldn’t answer. Her university did not allow her to use AI on any of her assignments and they did not integrate AI literacy classes or skills into any parts of the curriculum. For universities to stay relevant and to train students up for success, we must ensure that AI literacy is part of the educational process.

## Declaration of interests

The author declares no competing interests.

## References

[1] Anders B.A. (2023). Is using ChatGPT cheating, plagiarism, both, neither, or forward thinking?. Patterns.

[2] Gariepy L. (November 28, 2023). I’m a career expert: Here’s why you should put AI on your resume. Yahoo Finance.

[3] Gertenbach E. (February 23, 2024). Top AI skills to include on your resume, with examples. Upwork.

[4] Sanchez Y.W. (2023). The challenges and opportunities of AI-assisted writing: Developing AI literacy for the AI age. Technical Communication.

[5] Anders B. (2023).

[6] Kojima T., Gu S.S., Reid M., Matsuo Y., Iwasawa Y., Koyejo S., Mohamed S., Agarwal A. (2024). Proceedings of the 36th International Conference on Neural Information Processing Systems (NIPS ’22).

[7] Meskó B. (2023). Prompt engineering as an important emerging skill for medical professionals: tutorial. J. Med. Internet Res..

[8] Carucci R. (2024). https://www.forbes.com/sites/roncarucci/2024/02/06/in-the-age-of-ai-critical-thinking-is-more-needed-than-ever.

[9] Anders B. (2024).

